
JOURNAL INFORMATION
==============================
NLM Title Abbreviation: ScientificWorldJournal
Journal ID: ScientificWorldJournal
NLM Journal ID: 101131163
Journal ID: TSWJ

The Scientific World Journal

ISSN: 2356-6140
EISSN: 1537-744X
Publisher: Wiley

ARTICLE INFORMATION
==============================
PMCID: PMC5823088
PMID: 19838596
DOI: 10.1100/tsw.2009.123
Article ID: 895097
Article version: 1
Subjects: Clinical Images

Phlegmasia Cerulea Dolens — A Rare, Life-Threatening Condition

Vysetti S. *
Shinde S.
Chaudhry S.
Subramoney K.
Department of Internal Medicine, University of Illinois, Urbana-Champaign, IL, USA
*S. Vysetti: vysetti@uiuc.eduAcademic Editor: Daniel Batlle

Publication date: 2009
Electronic publication date: 2009 Oct 14
Volume: 9
First page: 1105
Last page: 1106
Received 2009 Sep 11; Accepted 2009 Sep 23
Copyright: Copyright © 2009 S. Vysetti et al.
Copyright year: 2009
License: This is an open access article distributed under the Creative Commons Attribution License, which permits unrestricted use, distribution, and reproduction in any medium, provided the original work is properly cited.
License URL: https://creativecommons.org/licenses/by/3.0/

Keywords: phlegmasia cerulea dolens

==============================
Clinical Image
*TheScientificWorldJOURNAL* (2009) 9, 1105–1106
ISSN 1537-744X; DOI 10.1100/tsw.2009.123

TheScientificWorldJOURNAL
www.thescientificworld.com

### Phlegmasia Cerulea Dolens – A Rare, Life-Threatening Condition

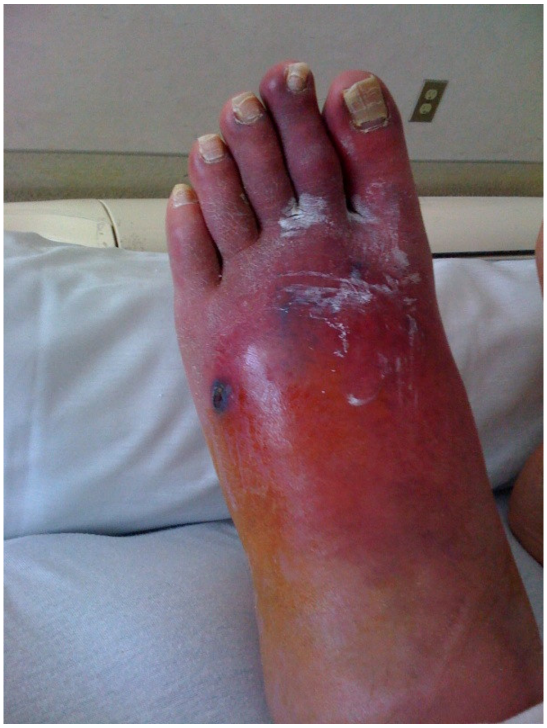

(A) At presentation

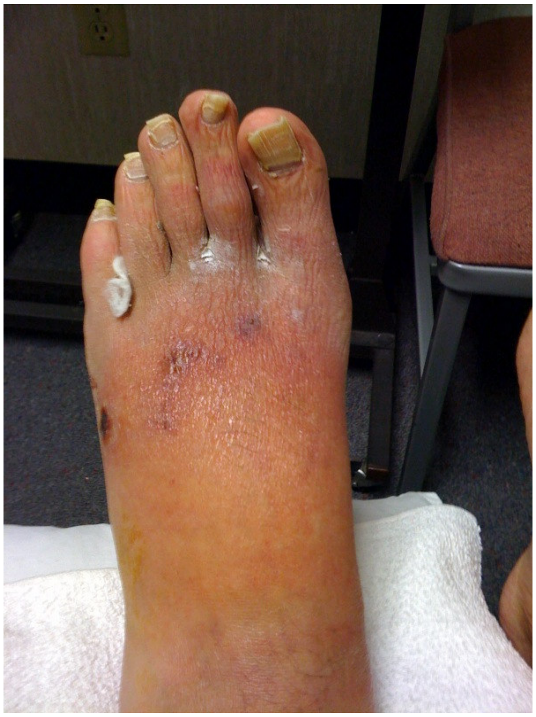

(B) Post-thrombectomy

S. Vysetti*, S. Shinde, S. Chaudhry, and K. Subramoney
*Department of Internal Medicine, University of Illinois, Urbana-Champaign*

**E-mail**: vysetti@uiuc.edu

*Received September 11, 2009; Accepted September 23, 2009; Published October 14, 2009*

**KEYWORDS**: phlegmasia cerulea dolens

Phlegmasia cerulea dolens is a rare form of massive proximal (e.g., iliofemoral) venous thrombosis of the lower extremities associated with a high degree of morbidity, presenting with sudden severe lower extremity pain with edema and cyanosis. Venous gangrene, venous congestion with massive fluid

*Corresponding author.
©2009 with author.
Published by TheScientificWorld; www.thescientificworld.com sequestration leading to circulatory collapse, and shock causing death can ensue if not promptly diagnosed and treated.

This entity was first described by Fabricus Hildanus in the 16th century. In 1938, Gregoire made an outstanding description of the condition and used the term "phlegmasia cerulean dolens" to differentiate ischemia-associated massive venous thrombosis from phlegmasia alba dolens, which describes fulminant venous thrombosis without ischemia[1]. The exact incidence of this condition is not reported. Precipitating factors include malignancy, which is the most common cause, femoral vein catheterization, heparin-induced thrombocytopenia, antiphospholipid antibody syndrome, surgery, heart failure, and pregnancy[2]. Pathophysiology includes extensive thrombosis involving the deep veins of the lower extremity, along with the collaterals leading to venous congestion with massive fluid sequestration and significant edema. Without established gangrene, this phase is reversible if proper measures are taken. Nearly 40–60% of the phlegmasia cerulea dolens cases also have capillary involvement, which results in irreversible venous gangrene that involves the skin, subcutaneous tissue, or muscle[1]. Under this condition, the hydrostatic pressure in arterial and venous capillaries exceeds the oncotic pressure, causing fluid sequestration in the interstitium and, if massive, can result in circulatory collapse[1].

There is no general consensus regarding the standard management. Traditionally, systemic anticoagulation and surgical thrombectomy has been the mainstay of treatment for this condition. More recently, systemic anticoagulation accompanied by pharmacomechanical catheter-directed deep venous thrombolysis is best indicated as first-line treatment for patients with phlegmasia cerulea dolens[3]. Multidisciplinary long-term clinical trials should validate the above technique in the future[3]. Prompt diagnosis and treatment initiation are paramount in order to prevent progression to venous gangrene and the need for amputation and possible death of the patient who presents with this condition.

We present a clinical image of a 55-year-old man with newly diagnosed cholangiocarcinoma who presented with worsening left lower extremity pain and swelling of 2-days duration. On examination, we found painful swelling with purple discoloration of the left foot (A) extending up to the ankle joint. Distal arterial pulses were palpable. Doppler ultrasound revealed extensive thrombosis in all segments of the deep vein bilaterally except the right iliac segment. Anticoagulation with heparin was initiated. The patient underwent an emergency left iliac thrombectomy for salvage of the limb and placement of a prophylactic inferior vena cava filter. The patient's symptoms resolved. The second image (B) is from a week after the procedure.

### REFERENCES

1. Dardik, A. and Rahhal, D. (2008) Phlegmasia alba and cerulea dolens. Emedicine. http://emedicine.medscape.com/article/461809-overview
2. Sarwar, S., Narra, S., and Munnir, A. (2009) Phlegmasia cerulea dolens. *Tex. Heart Inst. J.* **36(1),** 76–77.
3. Vedantham, S. (2008) Interventional approaches to acute venous thromboembolism. *Semin. Respir. Crit. Care Med.* **29(1),** 56–65.

**This article should be cited as follows:**

Vysetti, S., Shinde, S., Chaudhry, S., and Subramoney, K. (2009) Phlegmasia cerulea dolens – a rare, life-threatening condition. *TheScientificWorldJOURNAL* **9**, 1105–1106. DOI 10.1100/tsw.2009.123.
